# Psychological and Socio-Economical Determinants of Health: The Case of Inner German Migration

**DOI:** 10.3389/fpubh.2021.691680

**Published:** 2021-06-29

**Authors:** Bjarne Schmalbach, Ileana Schmalbach, Christoph Kasinger, Katja Petrowski, Elmar Brähler, Markus Zenger, Yve Stöbel-Richter, Ernst Peter Richter, Hendrik Berth

**Affiliations:** ^1^Medical Psychology and Medical Sociology, University Medical Center of the Johannes Gutenberg University Mainz, Mainz, Germany; ^2^Research Group Medical Psychology and Medical Sociology, Division of Psychological and Social Medicine and Developmental Neurosciences, Technische Universität Dresden, Dresden, Germany; ^3^Department of Psychosomatic Medicine and Psychotherapy, University Medical Center of the Johannes Gutenberg University Mainz, Mainz, Germany; ^4^Behavioral Medicine Research Unit, Integrated Research and Treatment Center Adiposity Diseases, Department of Psychosomatic Medicine and Psychotherapy, University of Leipzig Medical Center, Leipzig, Germany; ^5^Department of Applied Human Studies, University of Applied Sciences Magdeburg-Stendal, Stendal, Germany; ^6^Faculty of Managerial and Cultural Studies, University of Applied Sciences Zittau/Görlitz, Görlitz, Germany

**Keywords:** health, migration, socio-economic status, locus of control, German reunification, German migration

## Abstract

A substantial body of research has shown worse health conditions for East- vs. West-Germany in the wake of reunification. In the present study, we investigate how these differences between the two formerly divided regions developed and what maintains them. Specifically, we consider the associations between health status, income satisfaction, and health-related locus of control. In a quasi-experimental and longitudinal study design, we are particularly interested in the differences between individuals who stayed in East-Germany and those who were born in the East but migrated to West-Germany. To this end, we examined data from seven waves of the Saxony Longitudinal Study (2003–2009). Specifically, we tested a cross-lagged panel model with random effects, which evinced very good model fit. Most parameters and processes were equivalent between individuals who stayed in East-Germany vs. moved to West-Germany. Crucially, there was the expected pattern of positive correlations between health, income, and locus of control. In addition, we found substantially lower values for all three of these variables for the individuals who stayed in East-Germany (vs. moved to West-Germany). A possible explanation is the increase in socio-economic status that the internal migrants experienced. These findings present an important contribution of research in order to foster a better understanding on the social dynamics in Germany related to internal/domestic migrants and implications in the context of health outcomes (e.g., significantly more unemployment in East vs. West-Germany), especially since almost 20–25% of East-German citizens migrated to West-Germany. Until now, there are no similar studies to the Saxony longitudinal project, since the data collection started in 1987 and almost every year an identical panel has been surveyed; which can be particularly useful for health authorities. The study mainly focuses on social science research and deals with the phenomenon of reunification, approaching several subjects such as mental and physical health, quality of life and the evaluation of the political system. Yet even though many people have experienced such a migration process, there has been little research on the subjects we approach. With our research we deepen the understanding of the health consequences of internal migration.

## Introduction

The separation of Germany after the end of World War II and the subsequent existence of two German states—the German Democratic Republic (GDR; East Germany) and the Federal Republic of Germany (FRG; West Germany)—between 1949 and 1990 offers an opportunity to study the influence of factors such as socialization and social and political transformation on mental health. The distinct societies and cultures were not synchronized immediately in the course of reunification (1, 2). Instead, they assimilated over time (3, 4). With regard to citizens' health status in the formerly divided regions, many studies have shown that East-Germans were more at risk, when Germany was first reunited but assimilated over time (5–8). In this regard, there are several areas to consider, such as quality of life, psychological health, and job opportunities. However, the broad unemployment exemplifies its relevance in the German social system. In the course of German reunification, there were massive changes in the economy of East Germany. As a result, many people lost their jobs and had to reorient themselves professionally. To date, despite an economic upswing in recent years, the unemployment rates in the new federal states are about twice as high as in western Germany, which negatively affected the psychological and physical health of its citizens. For example, unemployed citizens reported significantly higher psychological stress, higher scores in depression, and lower quality of life than people who have never been unemployed. In addition, unemployment was significantly correlated with greater alcohol consumption, body weight, and somatic complaints (9–13), which on the long run significantly affect the overall health. Further studies replicated these results showing a higher psychological burden in citizens living in eastern Germany, compared to those living in West-Germany. Or to put it the other way around: People who have moved to the West were more relaxed and less stressed (14–16). What is more, it was suggested that specially the German population in East-Germany will shrink due to domestic migration to West-Germany (17).

Migration is usually associated with psychological and physical health difficulties and is mostly studied in the context of experiencing a different culture [e.g., language, food, etc.; (16)]. However, (18) reported greater depression in both, migrants from East to West-Germany as well as from West to East-Germany. Nevertheless, other studies reported greater quality of life in people who migrate from East to West-Germany (14, 15). Further, Albani et al. (19) showed a lower quality of life in citizens from West-Germany who migrate to East-Germany. On the other hand, even if the unemployment rate is still significantly higher in the east than in the west, a greater convergence can be observed after 30 years of reunification (20). For example, while the difference to the west was more than 10% points at the turn of the millennium, it was only around 2% points in 2018. This can be attributed to a positive development on the labor market and research into key technologies. Further, the development of living conditions in East and West since the fall of the Berlin Wall were no longer significantly different between the eastern and western federal states (20). Taking this background into consideration, a main questions that researchers are grappling with in this context is: Where did the differences in health between East- and West-Germany originate? And consequently, how can one explain the convergence that has been observed since reunification?

Health is a multiply determined construct (21, 22). In the present investigation, we combine psychological and socio-economic indicators to paint a complete picture as possible of the health development. First, we consider health-related locus of control (LoC). Decades of research have shown that the ability to control one's health, and—as if not more important—knowledge of and confidence in that ability are crucial determinants of health behavior and thus health in general (23). This confidence feeds on past achievements, and leads to a sense of control and a positive outlook which in turn results in more success. There is convincing evidence for the positive role of LoC—and its cousin self-efficacy (24, 25). Among others, both have been shown to be predictive of smoking cessation (26–28), nutrition, weight control (29, 30), and adherence to health programs and preventive health behaviors (31–35).

Second, we consider socio-economic status (SES) in the shape of satisfaction with one's income. Health-related outcomes are well-known to be determined by socio-economic factors and specifically the extent to which individuals have access to health care services (36, 37). Like any association, the factors influence each other. On the one hand, better health also offers better opportunities in life (38–40). For example, access to education is a positive factor and it is easier to find a well-paying job (41–44). On the other hand, a higher income is associated with better health. Through the higher income, one has better access to medical care, the possibility of an adequate nutrition and the opportunity to afford hygiene products and medical treatments (45–48). Finally, a higher income is to a substantial degree based on better education and thus a greater awareness of good health related behaviors (48–50).

Returning to the topic of questions that researchers are dealing with in the wake of reunification: A third question is inevitably intertwined with the second question mentioned above. That is, what role does the internal or domestic migration that took place following reunification (and still does to this day) play in this process? While Germany was divided as well as after the opening of the inner German border, a large number of East-Germans moved to the Western part of the country (16, 51, 52). Throughout the GDR's existence, around four million individuals fled the regime. After reunification, an average of 134 thousand East-Germans migrated to West-Germany each year, and only 89 thousand West-Germans moved to East-Germany. Over time, the gap narrowed, with comparable absolute numbers being reported from 2014 onward. Yet, when considering the smaller base population in East- vs. West-Germany, East-Germans are still much more likely to move to West-Germany than the other way around.

Based on previous research, we know that migration of all kinds is associated with stressful life events and acculturative stress (53–55). Psychosocial stress situations also arise where familiar habits are disturbed by social changes (56). In addition to the transformation experiences and reunification, changes of residence can also represent such a stressor. As mentioned above, millions of people moved from East Germany to West Germany because of the former established regime, which is a *push factor*. After the reunification and with the fall of the GDR, people moved for other reasons, such as better occupational opportunities and social structures, which are *pull factors* [e.g., (57)]. These may adversely affect the migrant's mental health and manifest themselves in somatic symptoms. At the same time, numerous studies have demonstrated a positive selection bias with regard to those who migrate for work-related reasons, known as the healthy migrant effect (58–60). In addition, it should be noted that a migration from East- to West-Germany (or vice-versa) is likely much less stressful than a “typical” migration, where an individual has to adapt to a completely different culture. Especially coupled with the fact that the migration to the West was largely perceived as an opportunity [e.g., better job and career perspectives; (14–16, 61), p. 15; (62, 63)], a positive effect of migration seems more likely (16, 64, 65).

For the study at hand, we are interested in internal/domestic migration in the wake of German reunification and how it affected individuals' health. To this end, we examined a longitudinal survey, the Saxony Longitudinal Study, which started collecting data even before the Fall of the Berlin Wall and followed its participants up until today (2020). We started our analysis in the 2003 wave, because by this point a respectable amount of internal migration had taken place, enabling reasonable statistical analyses. The last wave under consideration is 2009 because, starting here, there are gaps in the variables of interest, which until that point were collected on a yearly basis. Until now, there are no similar studies to the Saxony longitudinal project since the data collection has been going on for over 30 years; which can be particularly useful for health authorities.

We formulated the following hypotheses. We formulated the following hypotheses. (H_1_) First and foremost, we expect that our main outcome (health status) will be highly correlated with the two predictors (i.e., 1. income satisfaction and 2. health-related LoC).

(H_2_) Furthermore, in line with previous investigations (8), we assume that individuals in West-Germany (vs. East-Germany) will exhibit higher values of health status along with higher values for income satisfaction and LoC. On top of the general effects stated above, we hypothesize longitudinal dependencies in the auto-regressive latent trajectory model (66). Specifically, we expect that income satisfaction and LoC at Time x predict health at Time x+1 (H_3_ = cross-lagged effect). In addition, we postulate that health at Time x predicts health at Time x+1 (H_4_ = auto-regressive effect).

## Method

### Participants and Procedure

The sample at hand was collected within the scope of the Saxony Longitudinal Study (67). Beginning in 1987, a group of *N* = 1,407 8th graders at the time in East-German schools (Leipzig and Chemnitz—then Karl-Marx-Stadt—areas) has been assessed on a yearly basis with regard to political and health-related issues. In Wave 3 (1989), *N* = 587 individuals gave written consent for further participation in the study. In the seven survey waves of interest to the study at hand (2003–2009) *n* = 417 (71.0% of those who initially gave consent) individuals participated. However, 66 participants were missing more than half of the data points across time and were thus excluded. This resulted in a final sample of *n* = 351 participants. Of those, 266 remained in East-Germany throughout the time frame of analysis, whereas 85 migrated to West-Germany. Individuals that moved between 2003 and 2009 were not included in the analysis. The average age of the final sample was 30.03 (*SD* = 0.35) in 2003. We reported further sample details in Table 1.

**Table 1:** Sociodemographic variables of the sample (in 2003).

	***n*_**East**_**	**%_**East**_**	***n*_**West**_**	**%_**West**_**
Total	266	75.8	85	24.2
**Sex**				
Male	124	35.3	44	12.5
Female	142	40.5	41	11.7
**Family status**				
Unmarried, no partner	44	12.7	20	5.8
Unmarried, with partner	78	22.5	14	4.0
Civil union	43	12.4	14	4.0
Married	95	27.5	33	9.5
Divorced	4	1.2	1	0.3
**Employment status**				
Training	8	2.3	2	0.6
Employed	227	65.6	74	21.4
Unemployed	29	8.4	6	1.7

### Operationalization

We used the following three items to depict our main outcome variables for all seven measurement points:

*Current health status* was captured with the item “How would you describe your current health status?” The response options ranged from 1 (*bad*) to 5 (*very good*).

We inquired in to *satisfaction with one's income* among other issues. Respondents indicated their “Satisfaction with income” on a scale from 1 (*unsatisfied*) to 4 (*satisfied*).

Finally, we assessed *locus of control* (LoC) with the item “To what extent can you influence your own health status?” The response options ranged from 1 (*not at all*) to 5 (*very much*).

### Statistical Analyses

**Figure 1:**
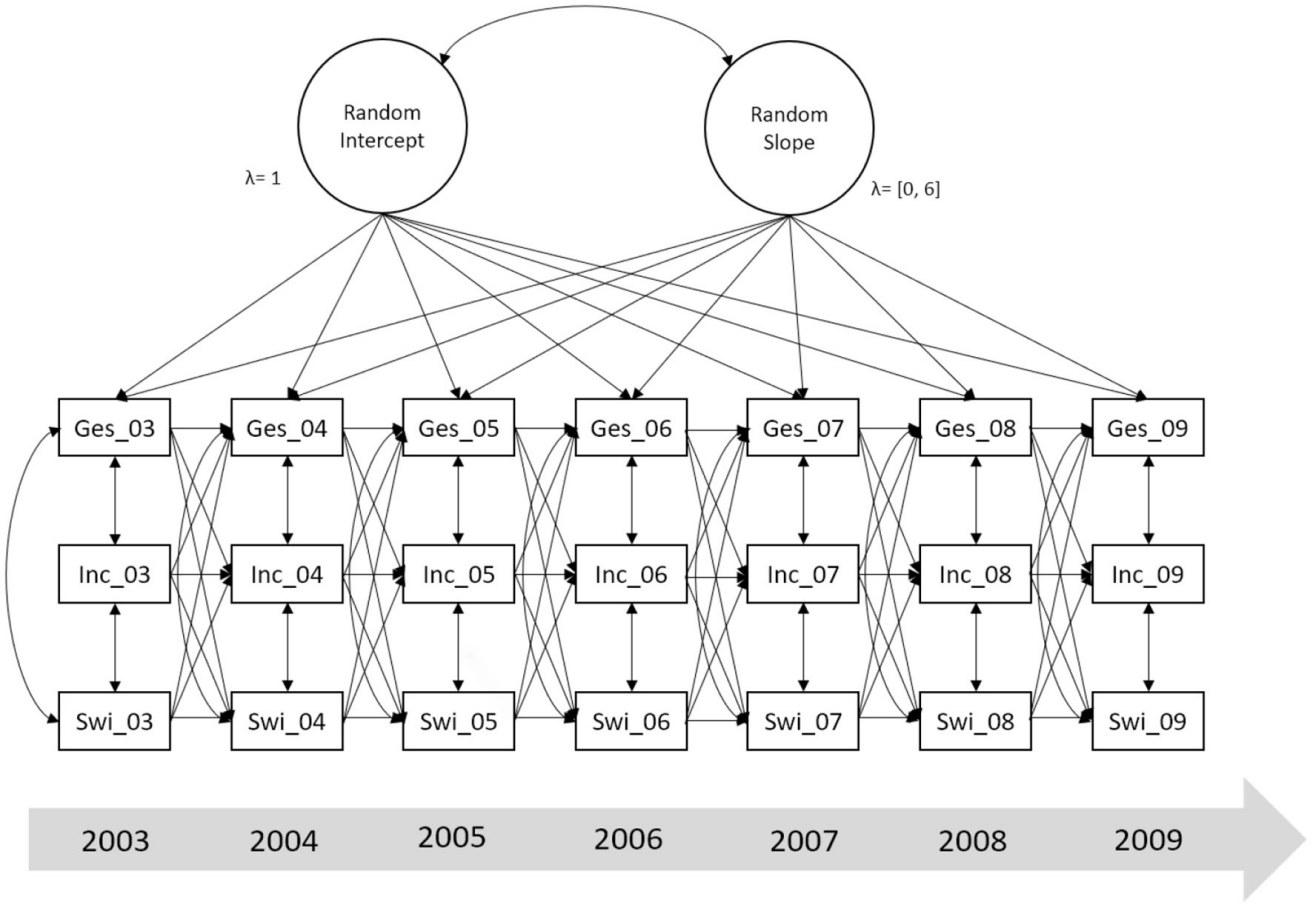
Simplified schematic of the autoregressive latent trajectory model of health status, income satisfaction, and locus of control. ges, subjective health status; inc, satisfaction with income; swirk, locus of control; 03–09, years. The diagram is simplified to facilitate understanding and interpretation. The actual model includes random intercepts and random slopes for all three constructs. Additionally, it includes phantom latent variables that act as go-betweens between the observed variables and the random intercepts and slopes. Furthermore, residual variances are not displayed.

We conducted all analyses in R, using the *bnstruct, ezCutoffs, ggcorrplot, ggplot2, Hmisc*, and *lavaan* packages (68–73). Since there were still 6.3% missing data across all measurement points, we imputed missing values using *k*-nearest-neighbor imputation. Initially, we calculated bivariate Pearson correlations between the three variables within and between all seven measurement points. For the main analysis, we then tested an autoregressive latent trajectory model with structured residuals (66). This model allowed us to differentiate within-subject and between-subject variation. The former will be captured by the classic cross-lagged parts of the model, whereas the latter will be captured in the random intercepts and slopes. Thus, the main advantage of this model is that it accounts for change as well as for stability. We modified the model suggested by Mund and Nestler by allowing the random intercept means to vary between groups and instead constraining the intercepts of the observed variables of the first measurement point to 0 in both groups. A simplified schematic of the model is displayed in Figure 1.

We estimated the model using the robust maximum likelihood method and evaluated it based on the scaled χ^2^ provided by Yuan and Bentler (74). In, addition to the χ^2^-test, we utilized the Comparative Fit Index (*CFI*), the Tucker-Lewis Index (*TLI*), the Root Mean Squared Error of Approximation (*RMSEA*), and the Standardized Root Mean Squared Residual (*SRMR*) to evaluate model fit. Here, we utilized the robust formulas to calculate *CFI, TLI*, and *RMSEA* (75, 76). We judged these indices based on the traditionally recommended cutoff values: the *p*-value of the χ^2^-test should be >0.05 (0.01), *CFI*/*TLI* >0.97 (0.95), RMSEA smaller than 0.05 (0.08), and SRMR smaller than 0.05 (0.10), respectively, to indicate good (acceptable) fit between theoretical model and empirical data (77). To supplement these traditional fixed cutoff values, we employed the simulation-based approach introduced by Schmalbach et al. (72). In the latter approach, the empirically derived fit values are compared to the fit index distributions obtained by simulating a large number of data sets with the same underlying parameter table and overall same properties as the empirical one. We then successively introduced parameter constraints, and evaluated them using the Δχ^2^-test.

## Results

### Baseline Comparisons and Descriptive Statistics

Because all individuals originally lived in East-Germany and remained there for a while after the start of the survey (1987), we have a quasi-experimental design. Nonetheless, we compared the baseline for the two subsamples of migrants and those who remained in East-Germany. For that purpose, we examined an earlier measurement point (Wave 10, in 1994) for the same individuals. Here, all respondents still resided in East-Germany. Unfortunately, health-related variables were only introduced in later waves of the survey, but we used a number of variables to capture the overall living conditions and thus examine whether any differences we may find later might be pre-existing. As can be seen in Table 2, there were no meaningful differences between any of the five variables. Overall, this evidence that individuals who stayed in East-Germany and those who later on moved to West-Germany were similar psychologically, at least prior to migration.

**Table 2:** Comparison of living conditions and life satisfactions at baseline (1994).

	***M*_**East**_**	***M*_**West**_**	***t***	***p***	***d***
Income satisfaction	2.47	2.42	−0.40	0.689	−0.060
Life situation	2.88	2.85	−0.25	0.803	−0.037
Life standard	3.17	3.19	0.20	0.844	0.031
Danger of personal distress	2.27	2.28	0.13	0.900	0.019
Confidence in future development	3.95	3.96	0.17	0.863	0.026

**Figure 2:**
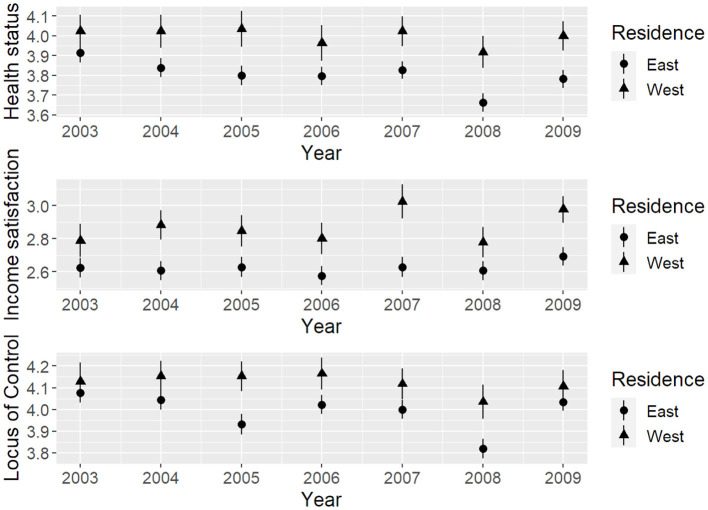
Observed mean scores of subjective health status, satisfaction with income, and locus of control for individuals in East- and West-Germany across time.

**Figure 3:**
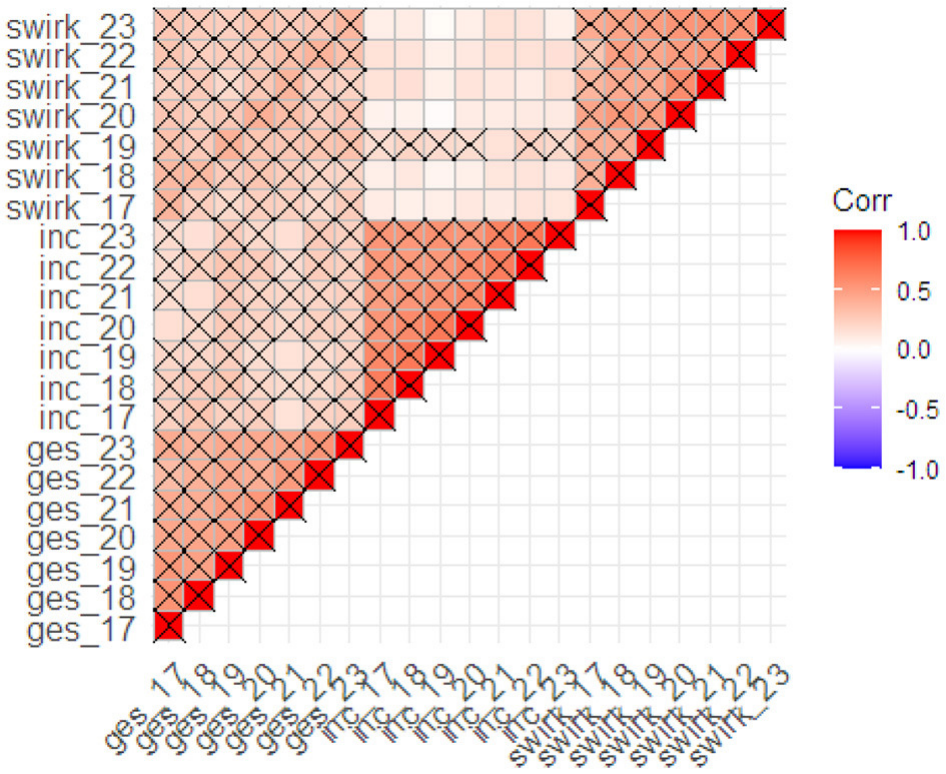
Observed correlations between the three dependent measures across all measurement points. ges, subjective health status; inc, satisfaction with income; swirk, locus of control; 17–23, survey wave (corresponding years: 2003–2009). Crossed cells are significant at *p* < 0.001.

We reported the distributions of the three variables of interest—subjective health status, satisfaction with income, and locus of control—across all seven measurement points in Table 3 and Figure 2. Next, as a rudimentary check of the associations between the observed variables, we calculated bivariate correlations across all measurement points (see Figure 3). Auto-regressions were significant with *r*s of 0.40–0.50 for health and LoC, and *r*s of 0.60–0.70 for income. Cross-correlations and cross-lagged correlations were slightly smaller. Health status correlated 0.20–0.30 with income at the same and the subsequent measurement points, and 0.30–0.40 with LoC. In contrast, correlations between income and LoC were comparatively smaller—in the 0.10–0.20 range.

**Table 3:** Descriptive statistics of subjective health status, satisfaction with income, and locus of control for individuals in East- and West-Germany across time.

	**Health status**		**Income satisfaction**		**Locus of control**	
	***M***	***SD***	***M***	***SD***	***M***	***SD***
**East-Germany (*****n*** **=** **266)**						
2003	3.91	0.77	2.62	0.96	4.08	0.72
2004	3.84	0.79	2.61	0.92	4.05	0.74
2005	3.8	0.8	2.63	1.00	3.93	0.78
2006	3.8	0.74	2.58	0.95	4.02	0.72
2007	3.83	0.73	2.63	0.97	4.00	0.71
2008	3.66	0.77	2.61	0.96	3.82	0.75
2009	3.78	0.72	2.69	0.9	4.03	0.63
**West-Germany (*****n*** **=** **85)**						
2003	4.02	0.77	2.79	0.93	4.13	0.80
2004	4.02	0.77	2.88	0.81	4.15	0.65
2005	4.04	0.82	2.85	0.88	4.15	0.63
2006	3.96	0.84	2.80	0.88	4.16	0.67
2007	4.02	0.69	3.02	0.95	4.12	0.66
2008	3.92	0.74	2.78	0.85	4.04	0.71
2009	4.00	0.69	2.98	0.74	4.11	0.69

### Longitudinal Analysis

The initial unconstrained model evinced very good fit, $\chi_{\left(348\right)}^{2}$ = 396.561, *p* = 0.037, *CFI* = 0.985, *TLI* = 0.982, *RMSEA* = 0.029, *SRMR* = 0.055. Goodness-of-fit was furthermore confirmed in the simulatory approach: Simulated cutoff values at α = 0.05 were χ^2^ = 428.533, *CFI* = 0.975, *TLI* = 0.970, *RMSEA* = 0.039, *SRMR* = 0.072. Thus, the empirical fit indices were never worse than the simulated cutoff values.

In order to compare the model between the two subsamples of individuals living in East- and West-Germany, we then introduced parameter constraints in a successive fashion. As can be seen in Table 4, the majority of parameters should be considered equal between the two groups. Only the random intercept means and time-point-specific cross-correlations differed substantially between groups. Specifically, the random intercepts for health status and income satisfaction were significantly higher for those respondents who had moved to West-Germany (see Table 5). A marginally significant effect was found for locus of control. The effect cross-correlations for the first measurement point were significantly higher for individuals in West- vs. East-Germany (see Table 6). The associated effect sizes were *q* = 0.282 and 0.247 for the difference in correlation coefficients between health status and income satisfaction, and health status and LoC, respectively.

**Table 4:** Nested model comparisons.

	**χ^**2**^**	***df***	**Δχ^2^**	**Δ*df***	***p***
Baseline	396.561	348			
Equal Random Effect (Co)Variances	408.668	369	12.107	21	0.937
Equal Phantom Latent Variable Variances	437.456	390	28.788	21	0.119
Equal Cross-lagged Effects	441.652	396	4.196	6	0.650
Equal Auto-regressive Effects	443.533	399	1.881	3	0.597
Equal Cross-correlated Effects	461.961	405	18.428	6	0.005^*^
Equal observed item intercepts	479.648	423	17.687	18	0.476
Equal Random Intercept Health	487.535	424	7.887	1	0.005^*^
Equal Random Intercept Income	486.630	424	6.982	1	0.008^*^
Equal Random Intercept LoC	483.449	424	3.801	1	0.051^†^

* *Significant at 0.05*.

† *Marginally significant at 0.10*.

**Table 5:** Random intercept means.

	***M*_**East**_**	***M*_**West**_**	***SD***	***d***
Health status	3.896	4.098	0.569	−0.354
Income satisfaction	2.606	2.857	0.725	−0.346
Locus of control	4.058	4.190	0.486	−0.271

*The mean of the random slope variables is 0 in both groups, per definition, and it is thus omitted. M, factor mean; SD, factor standard deviation*.

**Table 6:** Cross-correlation effects.

	**East**	**West**	***q***
Health status~~Income satisfaction 2003	−0.028	0.249	−0.282^*^
Health status~~Income satisfaction 2004	0.038	0.084	−0.046
Health status~~Income satisfaction 2005	0.035	0.077	−0.042
Health status~~Income satisfaction 2006	0.036	0.080	−0.044
Health status~~Income satisfaction 2007	0.037	0.081	−0.044
Health status~~Income satisfaction 2008	0.037	0.082	−0.045
Health status~~Income satisfaction 2009	0.049	0.107	−0.059
Health status~~Locus of control 2003	0.186	0.409	−0.247^*^
Health status~~Locus of control 2004	0.237	0.060	0.181^*^
Health status~~Locus of control 2005	0.218	0.055	0.166^*^
Health status~~Locus of control 2006	0.270	0.068	0.208^*^
Health status~~Locus of control 2007	0.283	0.072	0.219^*^
Health status~~Locus of control 2008	0.242	0.061	0.186^*^
Health status~~Locus of control 2009	0.354	0.090	0.281^*^
Income satisfaction~~Locus of control 2003	−0.028	−0.029	0.001
Income satisfaction~~Locus of control 2004	0.003	−0.101	0.104^*^
Income satisfaction~~Locus of control 2005	0.003	−0.089	0.092
Income satisfaction~~Locus of control 2006	0.004	−0.108	0.112^*^
Income satisfaction~~Locus of control 2007	0.003	−0.097	0.100
Income satisfaction~~Locus of control 2008	0.003	−0.097	0.100
Income satisfaction~~Locus of control 2009	0.005	−0.146	0.152^*^

*q, standardized difference between the two correlation coefficients in each row*.

* *Significant at 0.05*.

The following parameters were statistically equivalent between East and West and can thus be expected to function in comparable ways. First, the latent correlation matrix was equivalent (see Table 7): There were high correlations between the random intercept of health and income satisfaction, and health and LoC. In addition, there was a moderate correlation between income satisfaction and LoC. Furthermore, there was a significant correlation between the slopes of health status and income satisfaction. This means that as an individual's income increased so did their health and vice-versa. The same was not true for the slope of LoC.

**Table 7:** Random intercept and slope correlations.

		**Health status**		**Income satisfaction**		**Locus of control**	
		**Intercept**	**Slope**	**Intercept**	**Slope**	**Intercept**	**Slope**
Health status	Intercept	–					
	Slope	−0.489^*^	–				
Income satisfaction	Intercept	0.410^*^	−0.091	–			
	Slope	−0.226^*^	0.255^*^	−0.346^*^	–		
Locus of control	Intercept	0.542^*^	−0.085	0.241^*^	0.039	–	
	Slope	−0.041	−0.009	−0.134^*^	0.079	−0.137^*^	–

* *Significant at 0.05*.

As can be seen in Table 8, auto-regressive and cross-lagged effects did not reach a substantial magnitude for any of the associations (β < 0.10). Only the auto-regressions of income satisfaction reached βs of 0.20 and greater. Given our sample size, none of the effects (except for the auto-regression of income satisfaction) were significant. This implies that time point-specific variation in the variables explains only about 2–3% in health status in the next measurement. It should be noted, however, that this effect is controlled for the aforementioned effects of the random intercept correlations. This means that, in general across all time points, individuals with higher income satisfaction and a more internal locus of control had a better health status.

**Table 8:** Standardized auto-regressive and cross-lagged effects.

	**Health status**	**Income satisfaction**	**Locus of control**
Health status 2003 → 2004	0.075	0.043	0.049
Health status 2004 → 2005	0.081	0.045	0.051
Health status 2005 → 2006	0.085	0.047	0.063
Health status 2006 → 2007	0.090	0.043	0.059
Health status 2007 → 2008	0.077	0.042	0.050
Health status 2008 → 2009	0.093	0.052	0.069
Income satisfaction 2003 → 2004	0.029	0.212^*^	−0.005
Income satisfaction 2004 → 2005	0.027	0.191^*^	−0.004
Income satisfaction 2005 → 2006	0.029	0.204^*^	−0.006
Income satisfaction 2006 → 2007	0.032	0.188^*^	−0.005
Income satisfaction 2007 → 2008	0.032	0.219^*^	−0.005
Income satisfaction 2008 → 2009	0.033	0.234^*^	−0.006
Locus of control 2003 → 2004	0.037	−0.060	0.018
Locus of control 2004 → 2005	0.033	−0.051	0.015
Locus of control 2005 → 2006	0.036	−0.055	0.019
Locus of control 2006 → 2007	0.033	−0.042	0.015
Locus of control 2007 → 2008	0.032	−0.048	0.015
Locus of control 2008 → 2009	0.039	−0.060	0.021

* *Significant at 0.05*.

## Discussion

The aim of the present study was to investigate the health development of individuals in East- and West-Germany. Specifically, we compared a group of young adults that stayed in East-Germany vs. moved to West-Germany. First we hypothesized high correlations between (a) health and income satisfaction and (b) health and LoC. In addition, we assumed that individuals in West-Germany (vs. East-Germany) will exhibit higher values of health along with higher values for income satisfaction and LoC. Finally, we expected a cross-lagged and auto-regressive effects of the analyzed variables. For the main analysis, we tested an autoregressive latent trajectory model with structured residuals that enables to capture within-subject and between-subject variation. The model evinced very good fit, allowing us to conduct the examinations, we previously explained. As predicted, (H_1_) health status was strongly correlated with both, income satisfaction and LoC. These findings are in line with previous research demonstrating strong ties between health status, socio-economic status and self-regulation (23, 36, 37). Our second hypothesis (H_2_) was confirmed, indicating small- to moderately-sized effects in favor of the individuals that had moved to West-Germany (vs. had not moved) on all three of the variables of interest: Health, income satisfaction, health LoC. Yet, we found even larger effects in the correlations of the random intercepts, which is in line with previous analyses. Based on the overall analyses, this means that (irrespective of time) individuals with better SES and better LoC are also generally healthier. Our results emphasized that individuals with greater internal locus of control had a better health status, which corresponds with previous evidence on the subject (78–80). These studies also underlined the relevance of positive health attitudes, which tend to motivate individuals to behave in beneficial ways, rather opting for choices that promote health and at the same time reducing risks (81).

Concerning our last hypotheses (H_3_ and H_4_), our results point out a random slope of “0.” Thus, there is no general time-dependent change. Similarly, longitudinal effects (i.e., auto-regressive and cross-lagged effects) were small, and largely non-significant. The sole exception to this statement was the auto-regressive effect of income satisfaction. Finally, cross-correlative effects were also relatively small. The exceptions here are moderate correlations for health status with income satisfaction. These were also different between East- and West-German habitants. While the correlation was much larger for West-Germans at T1, it was close to zero after that, while remaining relatively constant for East-Germans. In sum this means, that the majority of systematic variance is found in general between-subject effects, namely in random intercept correlations and group differences. We observed little to no change over time. A possible explanation of these results could be that most individuals had already lived in their new residence for multiple years and were thus already habituated to the overall living conditions. Assuming equivalence prior to migration (which we showed for several central variables), this means that the East-West migrants assimilated to West-German levels of health and income rather quickly and stably. As previously reported (57), pull factors often play a decisive role in internal migration, which is also reflected in the study at hand in those people who moved from East to West (due the overall better living conditions). Additionally, the structures of the German social system that were established in Western Germany after World War II reflect a more open society, which possibly facilitated adaptation (82–84). Importantly, it should be noted that people who migrate due to occupational concerns are more likely to have positive attitudes, which reduces the stressful experience of relocation.

Overall, we suppose that the improvement in socio-economic standing is a likely cause for the difference that emerges between the two groups in our study. This assumption is based on the fact that West-Germany has had (and still has to this day) higher objective income levels than East-Germany (85). Higher income, better living conditions, and overall higher social security are known to improve health behaviors and related outcomes (36, 37, 49, 86). However, future studies are needed to confirm the interpretation of this data.

### Limitations

The present study suggested better health outcomes for the citizens living in West-Germany. Nevertheless, the results of the study at hand should be consider under the lens of the following limitations. First, a central limitation of the study is that we did not have information about respondents' health status prior migration to West-Germany. We approximated this variable by examining respondents' overall life satisfaction (among other variables). To ascertain our findings, future studies should aim to include pre-migration health status. Secondly, we focused exclusively on psychological indicators, which are based on the subjective perception of the respondents. Even for income, we used satisfaction with income, rather than the monetary income itself. Future research could complement our analyses by instead examining objective indicators of health and SES or combining subjective and objective variables. A further limitation represents the fact, that we investigated exclusively the migration processes from East- to West-Germany without comparing the migration processes from West-to East Germany. Future research may benefit from this comparison too.

Finally, we only analyze the changes in health status, income satisfaction, and locus of control by using one-item scales for each construct. We fully acknowledge the limited depth of the assessment that is offered by single-item measures, particularly for more complex constructs. However, in our case the criteria under investigation can easily be boiled down to a single sentence. It is very common for health status to be assessed with a singular item (87–89). Similarly, satisfaction with income (and other socio-economic variables) is very straightforward and has often been measured using short scales or even single items (90–92). For locus of control, it can be argued that this construct is more complex and should ideally be captured using multiple indicators. Overall, however, it should be noted that we did not rely on singular assessments for any of the three constructs. While we did only use one type of wording for each latent variable, each item was assessed on seven occasions leading to a highly reliable assessment for the random intercept and slope. Nevertheless, this procedure limits the validity of the results to a certain extent. It should also be noted however, that the repeated measures design (including seven timepoints) leads to a highly valid assessment when general processes across time are concerned (random intercept and slope). Even so, the findings should be replicated using more comprehensive, validated scales in the future.

## Conclusion

In the study at hand, we examined the health development of citizens in East and West-Germany. In general, our data suggested positive outcomes in individuals that moved to East-Germany compared to those who did not in terms of health, income satisfaction, and health LoC. We concluded that the East-West migrants assimilated to West-German levels of health and income rather quickly and stably. In this context, we reasoned that the better living conditions (e.g., pull factors, better income, open society, etc.) contributed to the differences between the two studied groups. In addition, other factors might have contributed to the favorable outcomes such as positive expectations about migrating to West-Germany. In this case having a buffering effect, making the adaptation process easier.

## Data Availability Statement

The datasets generated during and/or analysed during the current study are not publicly available, but are available from the corresponding author on reasonable request.

## Ethics Statement

Ethical review and approval was not required for the study on human participants in accordance with the local legislation and institutional requirements. The patients/participants provided their written informed consent to participate in this study.

## Author Contributions

BS: conceptualization, methodology, and writing of the main paper. IS: writing, introduction, discussion, review, and editing. KP and EB: software and validation. YS-R and HB: investigation. MZ, CK, and ER: visualization and editing. EB, KP, and HB: project administration. All authors contributed to the article and approved the submitted version.

## Conflict of Interest

The authors declare that the research was conducted in the absence of any commercial or financial relationships that could be construed as a potential conflict of interest.
